# Cross-national analysis of the risk factors of child malnutrition among children made vulnerable by HIV/AIDS in sub-Saharan Africa: evidence from the DHS

**DOI:** 10.1111/j.1365-3156.2011.02733.x

**Published:** 2011-02-09

**Authors:** Monica A Magadi

**Affiliations:** Department of Sociology, City UniversityLondon, UK

**Keywords:** child malnutrition, children made vulnerable by HIV/AIDS, risk factors, sub-Saharan Africa, multilevel logistic regression, Demographic and Health Surveys

## Abstract

**Objective:**

To examine the risk factors of malnutrition among children whose mothers are infected with HIV in sub-Saharan Africa (SSA).

**Methods:**

Multilevel logistic regression models applied to Demographic and Health Survey (DHS) data collected during 2003–2008 from 18 countries in SSA, where the DHS included HIV test data for adults of reproductive age.

**Results:**

Across countries in SSA, the risk of malnutrition among children whose mothers are infected with HIV is particularly high among children aged one, boys, multiple/twin births, those who were smaller than average at birth, or whose mothers had no education, or in poorest or single parent households. Although these risk factors generally apply to all children from the same communities, the higher risk of child malnutrition among those in the poorest households is amplified among children whose mothers are infected with HIV. Also, while in general children who are breastfed for up to 6 months are significantly less likely to be malnourished than those who were never breastfed; the benefit of breastfeeding is not evident among children whose mothers are infected with HIV.

**Conclusion:**

Contextual community/country HIV prevalences show interesting patterns: the risk of malnutrition among children whose mothers are infected with HIV is lower in countries with higher HIV prevalence. These findings have important implications for interventions to address malnutrition among children made vulnerable by HIV/AIDS in the SSA region.

## Introduction

The reduction in the spread of HIV/AIDS (Goal 6) and of under-five mortality (Goal 4) both feature prominently in the Millennium Development Goals (MDGs). Despite some progress being made towards both, HIV/AIDS continues to take a terrible toll and deaths of under-five children in sub-Saharan Africa (SSA) remain unacceptably high. Between 1990 and 2006, about 27 countries – the large majority in SSA (the region most adversely affected by HIV/AIDS) – made no progress in reducing childhood deaths (United Nations 2008). The WHO consultation on nutrition and HIV/AIDS in Africa recognized that ‘far-reaching steps need to be taken to reverse current trends in malnutrition, HIV infection and food insecurity in most countries in the region, in order to achieve the MDGs’ (WHO 2005). An improved understanding of the risk factors of malnutrition among vulnerable sub-groups of the population will help inform efforts to mitigate the impact of HIV/AIDS in the worst affected regions.

One way in which HIV/AIDS can adversely affect the health and nutritional status of children is through mothers’ infection. First, children may get infected with HIV from vertical mother-to-child transmission, which predisposes them to increased risks of malnutrition, ill-health, and mortality (Bunn 2009; Fergusson *et al.* 2009; Nalwoga *et al.* 2010). Second, children may be affected as a consequence of parents’ illness and death. Maternal survival and HIV infection are strong predictors of infant and child survival (Nakiyingi *et al.* 2003). The higher risk of under-five mortality among children born to HIV-positive women applies to all these children, including those who are not HIV-infected themselves (International HIV/AIDS Alliance 2003).

Besides higher mortality risk, modest increases in ill-health and malnutrition have been observed in orphans in the Demographic and Health Surveys (DHS) data, with maternal and double orphans being worst affected (Owen *et al.* 2009). Newborns whose mothers are infected with HIV have higher rates of foetal malnutrition than newborns of HIV seronegative mothers (Gangar 2009), and this disadvantage is likely to extend beyond infancy. A recent study of the household/community HIV/AIDS determinants of under-five child malnutrition in SSA identified children whose mothers were infected with HIV as the most vulnerable (Magadi 2010). In this paper, we did a cross-national analysis of the risk factors of malnutrition among this sub-group of children made vulnerable by HIV/AIDS in SSA. The specific objectives were to: determine the risk factors of malnutrition among children in SSA whose mothers are infected with HIV; establish the effect of contextual community/country HIV/AIDS factors on the risk of malnutrition among children whose mothers are infected with HIV; explore the community and country level variations in the risk of malnutrition among children whose mothers are infected with HIV; and compare the risk factors of malnutrition among children whose mothers are HIV positive, with the overall risk factors among all children in the same communities.

## Data and methods

### Data

The paper is based on secondary analysis of existing data from the international DHS programme from countries in SSA. It uses DHS data collected 2003–2008 from 18 countries in SSA where the DHS have included HIV testing among men and women of reproductive age. The availability of HIV test data from recent DHS provides a unique opportunity for population-based studies of factors associated with the HIV/AIDS epidemic in different contexts. The data analysed in this paper is based on children aged under five whose mothers are infected with HIV in households selected for HIV testing. A summary of the data analysed is given in Table 1.

**Table 1:** The sample of children whose mothers’ are HIV seropositive by country and age of child

	Childs age in completed years					
		
Country	0.00	1.00	2.00	3.00	4.00	Total cases
Burkina Faso 2003	7	3	12	8	7	37
Cameroon 2004	40	25	21	38	35	159
DR Congo 2007	4	9	12	8	4	37
Ethiopia 2005	8	14	18	9	16	65
Ghana 2003	11	11	12	10	12	56
Guinea 2005	3	8	5	4	3	23
Kenya 2003	37	24	25	39	25	150
Lesotho 2004/2005	68	58	55	65	63	309
Liberia 2007	10	13	12	14	9	58
Malawi 2004	48	50	34	53	45	230
Mali 2006	10	3	11	7	7	38
Niger 2006	2	9	1	8	3	23
Rwanda 2005	21	30	29	25	35	140
Senegal 2005	4	5	4	6	0	19
Sierra Leone 2008	3	5	5	8	2	23
Swaziland 2006	140	130	115	114	106	605
Zambia 2007	108	96	97	113	83	497
Zimbabwe 2005/2006	139	142	117	139	151	688
Total (sub-Saharan Africa)	663	635	585	668	606	3157

The distribution of the sample of children whose mothers are HIV seropositive (Table 1) suggests that individual country-level analysis is not possible because of inadequate sample size. In a number of countries (especially those with relatively low HIV prevalence such as Senegal, Sierra Leone, Guinea, Niger, Burkina Faso and DR Congo), the sample of under-five children whose mothers are infected with HIV is fairly small (i.e. <50) and inadequate for any meaningful individual country analysis. However, the standardized nature of the DHS sample design and data collection instruments makes it possible to pool data across countries to enable investigation into overall patterns across the SSA region. Pooling data across countries is necessary to achieve sufficient sample size and improved statistical power for detecting significant associations. The resulting hierarchical data structure (i.e. individuals nested within communities/clusters which are in turn nested within countries) requires specialized analytical techniques which are described in the next section.

### Methods of analysis

The analysis involves the application of multilevel regression models, necessary to adequately handle the hierarchical data structure resulting from the DHS cluster survey design and from pooling of datasets across countries, where individuals/households are nested within communities (clusters), which are in turn nested within countries. Traditional single-level regression models which assume independence of observations would not be appropriate for such data as the key independence assumption would be violated because of expected correlation between individuals within communities or countries. Multilevel analysis not only allows us to control for potential correlation of individuals within higher level units, but also enables us to estimate the extent to which individuals in the same community or country are correlated. The multilevel approach also allows us to examine contextual higher level risk factors (i.e. at community or country level), besides the individual or family level determinants.

The outcome variable of interest is child nutritional status (stunting, wasting and underweight). Anthropometric indicators of nutritional status (height-for-age, weight-for-age and weight-for-height) were used to define nutritional status of children. Those with a Z score <−2 were defined as undernourished: weight-for-age defining underweight; height-for-age defining stunting; and weight-for-height defining wasting. Thus, all our response variables constitute binary outcomes, taking a value of ‘1’ if a child is malnourished (i.e. stunted, wasted or underweight), and a value of ‘0’ otherwise.

A number of explanatory variables at individual child, family/household and contextual community/country levels, expected to be associated with child nutritional status are included in the analysis. These comprise the following:

- Child level characteristics (i.e. age of child, sex of child, birth order, multiple/twin birth, preceding birth interval, breastfeeding duration and size of child at birth);
- Maternal characteristics (i.e. mother’s age, educational attainment and single parenthood);
- Household and residence (household wealth index, orphanhood and urban/rural residence); and
- Contextual community/cluster- or country-level factors relating to HIV prevalence.

Multilevel modelling places particular emphasis on country and community variations in the risk of malnutrition among children whose mothers are infected with HIV, and the extent of clustering of undernutrition within countries and communities (clusters within country). The general form of the random intercepts multilevel logistic regression model used in the analysis may be expressed as follows:


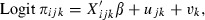
(1)

where π_*ijk*_ is the probability of being undernourished for a child *i*, in the *j*th community in the *k*th country; 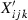 is the vector of covariates which may be defined at the individual, community or country level; β is the associated vector of usual regression parameter estimates; and the quantities *v*_*k*_ and *u*_*jk*_ are the residuals at the country and community level, respectively. These are assumed to have normal distribution with mean zero and variances 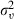 and 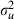 (Goldstein 2003).

The estimates of country and community level variances are used to calculate intra-unit correlation coefficients to examine the extent to which the risk of malnutrition among children whose mothers are infected with HIV is clustered within countries (or communities within countries) in SSA. The degree of clustering is measured before and after taking into account the effect of significant covariates. As children within the same community are also within the same country, the intra-community correlations include country variances (Siddiqui *et al.* 1996). Thus, the intra-community (ρ_*u*_) and intra-country (ρ_*v*_) correlation coefficients are given by:


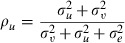
(2)

and


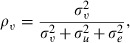
(3)

where 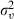 is the total variance at country level; 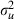 is the total variance at community level; and 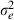 is the total variance at individual level.

For the multilevel logistic regression model, the level-1 residuals, *e*_ijk_, are assumed to have a standard logistic distribution with mean zero and variance π^2^/3, where π is the constant 3.1416 (Hedeker & Gibbsons 1996). The analysis was undertaken using MLwiN multilevel software, and estimations are based on second-order Predictive Quasi-Likelihood procedure (Rasbash *et al.* 2005).

Analysis started by examining the risk factors of malnutrition among children aged under five whose mothers are infected with HIV, before comparing the risk factors of malnutrition among children whose mothers are HIV positive with risk factors among all children observed in an earlier analysis (Magadi 2010).

## Results

### Risk factors of malnutrition among children aged under five with HIV-positive mothers

The bivariate distributions of malnutrition levels among children whose mothers are infected with HIV by individual/household characteristics are presented in the Appendix (Table A1). We recognize that the bivariate distributions presented in Table A1 do not provide precise risk factors for child malnutrition because the associations may be influenced by possible confounding factors associated with the risk of malnutrition. The results of the multilevel logistic regression models showing the independent risk factors of child malnutrition, while simultaneously controlling for the potential effects of other important factors, are presented in Table 2.

**Table 2:** Multilevel logistic regression parameter estimates of child malnutrition among children whose mothers are infected with HIV

Parameter	Stunted	Wasted	Underweight
*Fixed Effects*			
Constant	−0.14 (0.495)	−1.59 (0.824)	−0.61 (0.627)
Paternal orphan (non-orphan)			
Paternal orphan	−0.28 (0.155)	0.37 (0.284)	−0.32 (0.193)
Age of child (<1 year)			
1 year	1.49 (0.167)*	0.43 (0.262)	1.00 (0.204)*
2 years	1.01 (0.170)*	−0.41 (0.2994)	0.75 (0.209)*
3 years	1.11 (0.168)*	−0.76 (0.313)*	0.51 (0.210)*
4 years	0.62 (0.176)*	−1.22 (0.360)*	0.17 (0.222)
Sex of child (male)			
Female	−0.32 (0.086)*	−0.51 (0.170)*	−0.20 (0.106)
Birth order of child (fifth+)			
First birth	−0.25 (0.215)	−0.07 (0.394)	−0.45 (0.261)
Second	0.02 (0.166)	−0.09 (0.295)	−0.12 (0.202)
Third	−0.02 (0.157)	−0.57 (0.298)	−0.18 (0.190)
Fourth	−0.02 (0.161)	−0.60 (0.303)*	−0.24 (0.195)
Multiple/twin (no)			
Yes	1.10 (0.237)*	−0.18 (0.455)	1.44 (0.238)*
Birth interval (≤24 months)			
25–36 months	−0.14 (0.150)	0.19 (0.296)	−0.08 (0.185)
More than 36 months	−0.21 (0.148)	−0.32 (0.292)	−0.39 (0.177)*
Child breastfed (never)			
Upto 6 months	−0.41 (0.325)	−0.58 (0.478)	−0.40 (0.450)
More than 6 months	0.27 (0.286)	−0.58 (0.426)	0.39 (0.393)
Size of child at birth (small)			
Average	−0.57 (0.124)*	−0.34 (0.227)	−0.38 (0.142)*
Larger than average	−0.81 (0.134)*	−0.51 (0.247)*	−0.96 (0.161)*
Residence (urban)			
Rural	0.19 (0.127)	−0.29 (0.228)	−0.13 (0.156)
Mother’s education (none)			
Primary	−0.09 (0.143)	−0.03 (0.254)	−0.25 (0.165)
Secondary+	−0.37 (0.161)*	0.17 (0.281)	−0.34 (0.190)
Mother’s age (15–19)			
20–24	−0.44 (0.269)	0.05 (0.518)	−0.27 (0.321)
25–29	−0.68 (0.282)*	0.57 (0.528)	−0.30 (0.336)
30–34	−0.40 (0.297)	0.79 (0.554)	−0.15 (0.355)
35+	−0.56 (0.319)	0.50 (0.591)	−0.32 (0.382)
Single parent (no)			
Yes	0.30 (0.109)*	0.11 (0.212)	0.26 (0.134)
Wealth index (poorest)			
Poorer	−0.27 (0.137)*	0.14 (0.271)	−0.39 (0.162)*
Middle	−0.49 (0.142)*	−0.06 (0.276)	−0.38 (0.164)*
Richer	−0.31 (0.151)*	−0.04 (0.291)	−0.47 (0.180)*
Richest	−0.82 (0.181)*	−0.63 (0.347)	−1.30 (0.231)*
*Contextual factors*			
HIV prevalence in community	0.16 (0.232)	−0.22 (0.443)	−0.10 (0.284)
HIV prevalence in country	−0.22 (0.878)	−2.94 (0.857)*	−2.61 (0.999)*
*Random effects*			
Cluster – constant	0.33 (0.099)*	0.26 (0.313)	0.27 (0.135)*
Country – constant	0.10 (0.055)	0.00 (0.000)	0.12 (0.069)

* Statistical significance at 5% level –*P* < 0.05.

The results suggest that across countries in SSA, the risk of malnutrition among children whose mothers are HIV positive varies significantly by child’s age, sex, multiple/twin birth and size of child at birth. The risks of stunting and underweight are highest among children aged one, male children, multiple/twin births or those who were small at birth, while lowest for infants, girls, singletons and those who were bigger than average at birth. The pattern for wasting tends to follow a similar pattern for most of the indicators (except child’s age), but the associations tend to be weaker. Unlike stunting and underweight where the lowest risk is observed among infants, the risk of wasting is lowest among the older children aged four. Also, the risk of wasting tends to vary by birth order, being highest among births of order five and above.

The risk of child undernutrition by mother’s or household characteristics is generally consistent with patterns observed in the bivariate distributions. On average, children of teenage mothers or those whose mothers have no education, or who live in poorer or single parent households have higher risks of stunting. However, the risk of wasting and underweight are not significant by mothers’ age, educational attainment or single parenthood status. Also, greater household wealth is associated with reduced risk of stunting or underweight, but it is not significant for the risk of wasting.

Although higher HIV prevalence in communities or countries might be expected to be associated with a higher risk of malnutrition because of the impoverishing effects of HIV/AIDS in communities, it is interesting to note that the risk of undernutrition is generally lower in communities or countries with higher HIV prevalence. Overall, the evidence presented in Table 3 provides no evidence of increased vulnerability among paternal orphans, or among children in communities or countries with higher HIV prevalence. In fact, there is some indication that children of HIV-positive mothers living in countries with higher HIV prevalence have a lower risk of wasting or underweight, compared to their counterparts of similar characteristics in lower HIV prevalence countries.

**Table 3:** Average odds ratios of malnutrition for all children and those whose mothers are HIV positive

	Stunted		Wasted		Underweight	
			
Parameter	All	Mum+	All	Mum+	All	Mum+
Household HIV status (none+)						
Mother HIV+	1.28*	–	1.26*	–	1.26*	–
Other adults HIV+	0.97	–	1.03	–	0.97	–
Paternal orphan (non-orphan)						
Paternal orphan	0.86*	0.76	0.90	1.45	0.80*	0.73
Age of child (<1 year)						
1 year	3.63*	4.44*	1.19*	1.54	2.18*	2.72*
2 years	3.25*	2.75*	0.55*	0.66	1.82*	2.12*
3 years	3.39*	3.03*	0.38*	0.47*	1.36*	1.67*
4 years	3.22*	1.86*	0.33*	0.30*	1.27*	1.19
Sex of child (male)						
Female	0.80*	0.73*	0.82*	0.60*	0.88*	0.82
Birth order of child (fifth+)						
First birth	0.66*	0.78	0.92	0.93	0.69*	0.64
Second	0.83*	1.02	0.87	0.91	0.81*	0.89
Third	0.90*	0.98	0.87*	0.57	0.89*	0.84
Fourth	0.92*	0.98	0.94	0.55*	0.97	0.79
Multiple/twin (no)						
Yes	2.12*	3.00*	1.22*	0.84	2.03*	4.22*
Birth interval (24 months or less)						
25–36 months	0.86*	0.87	0.94	1.21	0.89*	0.92
More than 36 months	0.74*	0.81	0.99	0.73	0.76*	0.68*
Child breastfed (never)						
Upto 6 months	0.47*	0.66	0.44*	0.56	0.22*	0.67
More than 6 months	1.19*	1.31	0.97	0.56	1.13	1.48
Size of child at birth (small)						
Average	0.74*	0.57*	0.76*	0.71	0.63*	0.68*
Larger than average	0.63*	0.44*	0.59*	0.60*	0.46*	0.38*
Residence (urban)						
Rural	1.32*	1.21	0.88*	0.75	1.16*	0.88
Mother’s education (none)						
Primary	0.86*	0.91	0.88*	0.97	0.79*	0.78
Secondary+	0.68*	0.69*	0.76*	1.19	0.58*	0.71
Mother’s age (15–19)						
20–24	0.85*	0.64	1.17	1.05	0.90	0.76
25–29	0.78*	0.51*	1.07	1.77	0.84*	0.74
30–34	0.73*	0.67	1.12	2.20	0.79*	0.86
35+	0.68*	0.57	1.13	1.65	0.79*	0.73
Single parent (no)						
Yes	1.14*	1.35*	1.08	1.12	1.25*	1.30
Wealth index (poorest)						
Poorer	0.92*	0.76*	0.97	1.15	0.90*	0.68*
Middle	0.88*	0.61*	0.87*	0.94	0.85*	0.68*
Richer	0.78*	0.73*	0.82*	0.96	0.74*	0.63*
Richest	0.53*	0.44*	0.65*	0.53	0.50*	0.27*

Reference categories given in brackets.

* Significant at 5% level (*P* < 0.05).

The results provide evidence of significant variations in the risk of stunting and underweight among children of HIV-positive mothers across communities in SSA, even after taking into account a range of individual/household and contextual factors included in the model. The estimates of intra-unit correlations suggest that slightly more than 10% of the total unexplained variation in stunting and underweight among these children is attributable to unobserved community factors.

### Comparing children whose mothers are HIV positive with all children from the same communities in SSA

In this section, we focus on the vulnerability of children whose mothers are infected with HIV by comparing the risk factors of undernutrition among this particular sub-group of children with the risk factors among all under-five children in the same communities and countries in SSA (Magadi 2010). A comparison of the results of the multilevel logistic regression models for risk factors of stunting, wasting and underweight is presented in Table 3. Overall, fewer factors are significant for children whose mothers are HIV seropositive, presumably because of the considerably smaller sample size, reducing our statistical power to detect significant associations, but the patterns of the associations tend to be in the same direction as for all children.

**Figure 1:**
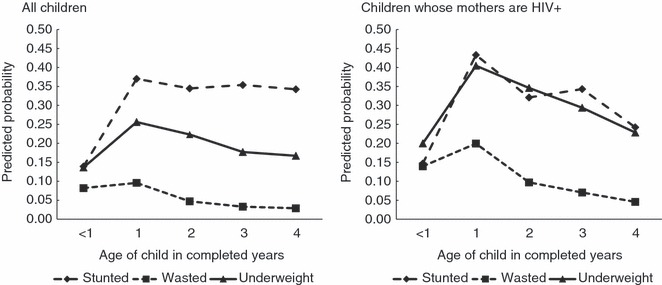
Predicted probabilities of malnutrition by age of child (derived from estimates in Table 3).

Similar to the patterns for all children, the risk of stunting or underweight among children whose mothers are HIV positive is lowest in infants, while the risk of wasting is lowest among the older children aged 4 years (Figure 1). For all the three indicators of undernutrition, the risk is highest among children aged one. The peak malnutrition rates at age one, with subsequent decreases at higher ages, may be attributable to early death of the most malnourished. The peak is likely to be especially sharp for the HIV-exposed children because HIV-infected children are both more likely to be malnourished and more likely to die very young.

The gender disparity in wasting appears greater among children whose mothers are infected with HIV (Table 3). On average, girls whose mothers are infected with HIV have 40% lower odds of wasting than boys with similar characteristics (among all children, the risk for girls is only 18% lower than for boys). Also, the higher risk of stunting or underweight among multiple births is amplified among children whose mothers are infected with HIV. For instance, multiple births have, on average, about double the odds of being underweight as singleton births – among children whose mother are HIV positive, the risk of being underweight among multiple births is about four times higher.

It is interesting to note that although children who are breastfed for up to 6 months are significantly less likely to be undernourished based on all the three indicators than children who were never breastfed, there is no evidence of a significant difference between children breastfed and never breastfed children among those whose mothers are HIV seropositive.

**Figure 2:**
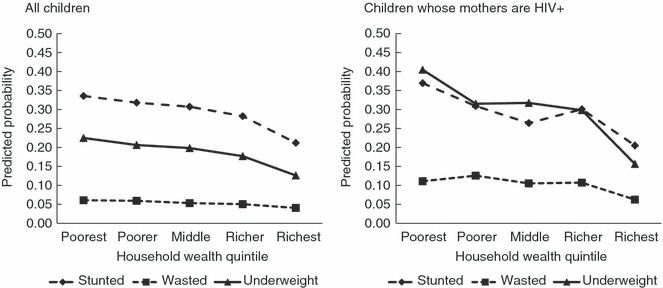
Predicted probabilities of child malnutrition by household wealth quintile.

The risk of undernutrition by mother’s or household characteristics shows generally similar patterns among children whose mothers are HIV seropositive and all children, but the higher risk among children in lower socio-economic sub-groups appears amplified among those whose mothers are HIV positive. In particular, children of HIV-positive mothers in the poorest quintile households have almost four times the odds of being underweight as children of similar characteristics in the richest quintile households, while among all children the odds among those in the poorest quintile households are double the odds of those from the richest household quintile. Estimates of predicted probabilities^1^ presented in Figure 2 suggest that there is little difference between children whose mothers are HIV positive and all children among those living in the richest households.

**Figure 3:**
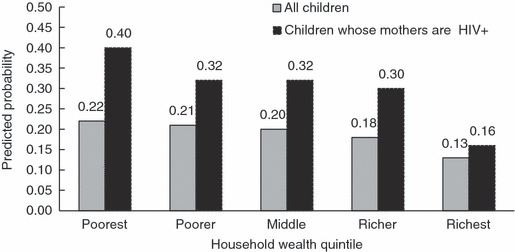
Predicted probabilities of underweight by household wealth quintile.

However, children whose mothers are infected with HIV are particular disadvantaged among those in the poorest household quintile, especially with respect to underweight (Figure 3). For instance, among children in the poorest households, the predicted probability of being underweight is about 40% among children whose mothers are HIV seropositive, compared to 22% among all children. The gap between children whose mothers are HIV seropositive and all children is much smaller among those in the richest households – 16% among children whose mothers are HIV seropositive and 13% among all children from the same communities/countries.

## Discussion and conclusions

Overall, the results suggest a particularly high risk of malnutrition among children aged one, especially with respect to stunting. On average across countries in SSA, children aged one whose mothers are HIV seropositive have more than four times the odds of being stunted as their younger counterparts of similar characteristics aged under one. The risk factors with respect to other child level characteristics largely conform to what might be expected, showing a higher risk of malnutrition among multiple/twin births, children who were very small or smaller than average at birth and male children. These risk factors apply to all children from the same communities/countries.

However, it is important to note that while in general children who are breastfed for up to 6 months are significantly less likely to be malnourished than those who were never breastfed, the benefit of breastfeeding is not evident among children whose mothers are infected with HIV. This finding has important implications for breastfeeding guidelines for HIV-seropositive mothers in SSA. The latest (2010) WHO guidelines recommend that ‘mothers known to be HIV-infected (and whose infants are HIV uninfected or of unknown HIV status) should exclusively breastfeed their infants for the first 6 months of life, introducing appropriate complementary foods thereafter, and continue breastfeeding for the first 12 months of life’, while being on long Anti-retroviral therapy (ART) regimen during the breastfeeding period (WHO 2010). In SSA setting where exclusive breastfeeding is often not practical for a variety of reasons (Shankar *et al.* 2005; Buskens *et al.* 2007) and few mothers receive the recommended ART regimens (WHO/UNAIDS/UNICEF 2009), the risk of mother-to-child transmission of HIV during breastfeeding remains a crucial issue.

With respect to mother’s or household socio-economic risk factors, the results conform to what might be expected showing a lower risk of malnutrition among children from more affluent sub-groups of the population such as those whose mothers have higher educational attainment or from richer households, consistent with findings from previous studies (Griffiths *et al.* 2004; Smith *et al.* 2005; Boyle *et al.* 2006; Fotso 2007). Although the overall patterns are similar to those for all children, the effect of household wealth is stronger among children whose mothers are HIV seropositive, especially the risk of underweight. The fact that children whose mothers are HIV seropositive are particularly vulnerable if they are living in poor households calls for targeted interventions to address overall malnutrition among this special sub-group of children.

It is interesting to note that there is a lower risk of malnutrition among children whose mothers are HIV positive living in countries with higher HIV prevalence, compared to countries with lower prevalence. It is possible that countries in the advanced stages of the HIV/AIDS epidemic are already making progress to put in place appropriate measures to mitigate the adverse impact of HIV/AIDS on vulnerable members of their populations. However, those in the early stages of the epidemic are more likely to face stigma and discrimination challenges, thwarting efforts to address the HIV/AIDS impact among minority vulnerable sub-groups of the population. Another possible explanation for the unexpected result showing lower levels of undernutrition in communities and countries of higher HIV prevalence may be attributable to possible compounding by other factors associated with both malnutrition and HIV prevalence. For instance, undernutrition is generally less common among the more affluent sub-groups of the population such as urban residents and wealthier households or countries, factors also known to be associated with higher HIV prevalence (Magadi & Desta 2009; Mishra *et al.* 2007). Although both urban/rural residence and household wealth status were included in the multivariate model, it is possible that the multivariate analysis may not have entirely controlled for these effects, given the crude nature of indices used.

There are significant variations in the risk of malnutrition among children whose mothers are HIV seropositive across communities in SSA, even after taking into account important child, mother and household level background characteristics, in addition to contextual factors at community/country level relating to HIV prevalence. More than 10% of the total unexplained variation in the risk of stunting and underweight is attributable to unobserved community level factors which may include traditional child feeding practices which are likely to vary across communities and different ethnic groups. Any interventions to address the problem of malnutrition among children of HIV-seropositive mothers in SSA should be sensitive to prevailing child feeding practices in the local communities.

## Footnotes

1 The predicted probabilities are derived while holding the other factors in the model at their mean values.
